# Brain injury biomarkers and intraoperative hypotension: associations with pituitary hormone deficiency following transsphenoidal endoscopic surgery for non-functioning pituitary adenomas

**DOI:** 10.1007/s11102-025-01597-y

**Published:** 2025-12-08

**Authors:** Martin Thorsson, P. Trimpou, M. Asztély, T. Hallén, V. Hantelius, K. Blennow, H. Zetterberg, G. Johannsson, J. Oras, T. Skoglund

**Affiliations:** 1 Department of Anesthesiology and Intensive Care, Institute of Clinical Sciences, The Sahlgrenska Academy, University of Gothenburg, Gothenburg, Sweden; 2 Department of Anesthesiology and Intensive Care, Sahlgrenska University Hospital, Gothenburg, Region Västra Götaland, Sweden; 3 Department of Internal Medicine and Clinical Nutrition, Institute of Medicine, The Sahlgrenska Academy, University of Gothenburg, Gothenburg, Sweden; 4 Department of Endocrinology, Sahlgrenska University Hospital, Region Västra Götaland, Gothenburg, Sweden; 5 Northern Älvsborg County Hospital, Trollhättan, Region Västra Götaland, Sweden; 6 Department of Clinical Neuroscience, Institute of Neuroscience and Physiology, The Sahlgrenska Academy, University of Gothenburg, Gothenburg, Sweden; 7 Department of Neurosurgery, Sahlgrenska University Hospital, Gothenburg, Region Västra Götaland, Sweden; 8 Department of Psychiatry and Neurochemistry, Institute of Neuroscience and Physiology, The Sahlgrenska Academy, University of Gothenburg, Gothenburg, Sweden; 9 Clinical Neurochemistry Laboratory, Sahlgrenska University Hospital, Gothenburg, Region Västra Götaland, Sweden; 10 Department of Neurodegenerative Disease, UCL Institute of Neurology, Queen Square, London, UK; 11 UK Dementia Research Institute at UCL, London, UK; 12 Hong Kong Center for Neurodegenerative Diseases, Clear Water Bay, Hong Kong, China; 13 Wisconsin Alzheimer’s Disease Research Center, School of Medicine and Public Health, University of Wisconsin, Madison, WI USA

**Keywords:** Postoperative complications, Pituitary neoplasms, Neurofilament protein, Glial fibrillary acidic protein, Tau protein, Hypotension

## Abstract

**Purpose:**

Factors related to the development of new pituitary hormone deficiencies following transsphenoidal surgery for non-functioning pituitary adenomas are multifactorial and remain poorly understood. We explored associations with brain injury biomarkers and investigated intraoperative hypotension (IOH) as a potential mediator.

**Methods:**

This prospective study included 100 patients undergoing endoscopic transsphenoidal surgery. Two individual outcomes at 12-months postsurgery were analysed: new anterior pituitary hormone deficiency (APH-D) and new arginine vasopressin deficiency (AVP-D). Plasma concentrations of glial fibrillary acidic protein (GFAP), neurofilament light chain (NfL), and tau were measured preoperatively and on postoperative days 1 and 5. IOH was assessed using two definitions: duration below an absolute MAP threshold of 65 mmHg and duration below a relative threshold of 20% below preoperative MAP. Associations between new deficiency and biomarkers were assessed using mixed-effects models, and associations with IOH were evaluated using the Mann–Whitney *U* test.

**Results:**

Elevated postoperative GFAP, NfL, and tau were associated with new APH-D, with GFAP also linked to new AVP-D. Patients who experienced new APH-D demonstrated longer durations of relative IOH (median [IQR] 155 min [54–216] vs. 82 min [20–154]; *p* = 0.03). There was no difference in relative or absolute IOH for those with new AVP-D.

**Conclusion:**

Elevated postoperative plasma GFAP, NfL, and tau might indicate increased risk of long-term postoperative pituitary hormone deficiency. Relative IOH may also contribute to these deficiencies.

**Supplementary Information:**

The online version contains supplementary material available at 10.1007/s11102-025-01597-y.

## Introduction

Transsphenoidal surgery remains the standard treatment for non-functioning pituitary adenomas (NFPAs) to alleviate symptoms and reduce tumour burden while preserving or restoring hormone function. Despite advances in surgical techniques, new postoperative pituitary hormone deficiencies remain a significant concern, occurring in up to 30% of cases [1], and potentially contributing to long-term morbidity and mortality [2, 3].

Identifying factors associated with new deficiencies may provide mechanistic insights, inform risk-mitigation strategies, and enhance surveillance methods for individuals at risk. However, only a few risk factors have been established to date. Current evidence points to tumour size, surgeon experience, postoperative cortisol concentrations, and sodium levels in cases of arginine vasopressin deficiency (AVP-D; formerly diabetes insipidus) as relevant risk factors [4–7].

Circulating brain injury biomarkers are proteins or protein fragments released into the bloodstream in response to neuronal or astroglial injury and detectable via blood test. In recent years, these biomarkers have been investigated as tools for assessing brain injury following neurosurgical procedures [8]. Among them, elevated glial fibrillary acidic protein (GFAP), neurofilament light chain (NfL), and tau concentrations have been identified following endoscopic transsphenoidal surgery (ETS), with tau also associated with postoperative fatigue [9]. Additionally, both GFAP and NfL have been linked to adverse outcomes after other types of neurological surgery, suggesting their potential efficacy for predicting clinical outcomes [10].

Elevated postoperative NfL and tau are also associated with intraoperative hypotension (IOH) during ETS [11]. This suggests that IOH during ETS could contribute to inadequate perfusion of vulnerable neural structures during surgical manipulation, and that IOH may represent a novel, potentially modifiable risk factor for new postoperative pituitary hormone deficiencies.

This study tested the hypothesis that new anterior pituitary hormone deficiency (APH-D) or new AVP-D at 12-months postsurgery was associated with elevated postoperative circulating GFAP, NfL, and tau, as well as IOH.

## Methods

### Study design and participants

The study included individuals aged ≥18 years with NFPA, undergoing ETS between 2016 and 2021, and enrolled in the prospective Gothenburg Pituitary Tumor study (GoPT). The study was registered on researchweb.org (https://www.researchweb.org/is/sverige/project/161671). All participants provided written informed consent. The study was conducted in accordance with the Declaration of Helsinki and approved by the Regional Ethics Review Board in Gothenburg, Sweden (Dnr: 387 − 15, 6/20/2015).

### Surgical procedures

Standard intraoperative monitoring techniques, including invasive blood pressure measurement, were used throughout all surgeries. General anesthesia was initiated with a bolus of propofol and infusion of remifentanil or a bolus of fentanyl, and maintained with either remifentanil infusion or fentanyl boluses along with inhaled sevoflurane or propofol infusion. Mean arterial pressure (MAP) was targeted at least above 65 mmHg and, in most cases, maintained above 70 mmHg. Blood pressure was supported using intravenous boluses of ephedrine, phenylephrine, or a continuous norepinephrine infusion. All patients received standardized perioperative hydrocortisone supplementation: 100 mg twice on the day of surgery, starting before surgery, and 50 mg twice on the first postoperative day.

### Outcomes

At 12-months postsurgery, new anterior pituitary hormone deficiencies (APH-D; corticotropic or thyrotropic) and new AVP-D were assessed as two separate outcomes. This follow-up time was chosen because deficiencies persisting at this time point are less likely to recover and thus provide a more clinically meaningful and robust assessment than evaluations performed at earlier postoperative time points [12, 13]. Participants with pre-existing deficiencies in both corticotropic and thyrotropic hormones or AVP-D were excluded from the respective outcome analyses. Gonadotropic deficiency was not assessed due to the high proportion of postmenopausal women in the cohort, and somatotropic deficiency was not routinely evaluated before surgery.

To assess corticotropic deficiency, morning serum cortisol levels were measured. Values > 350 nmol/L were considered normal, whereas values < 100 nmol/L were regarded as insufficient. Patients with inconclusive function underwent further testing with an adrenocorticotropic hormone test according to published guidelines [14]. Thyrotropic hormone deficiency was evaluated based on low free thyroxine levels and low thyroid-stimulating hormone levels. A diagnosis of postoperative AVP-D required polyuria levels > 2.5 L over a 24-h period, report of increased thirst, and low urine specific gravity (< 1.005), high serum sodium (> 145 mmol/L), or high serum osmolality (> 300 mOsm/kg), with subsequent treatment using desmopressin resulting in symptom resolution and normalization of serum osmolality and serum sodium [15]. A water-deprivation test was performed in the event of an uncertain diagnosis [16]. If the patient was on replacement therapy before surgery, no new testing was performed, and that axis was classified as deficient at baseline. Postoperative initiation of replacement therapy for corticotropic deficiency and AVP-D was re-evaluated at 12-months postsurgery using the same criteria as at baseline. If thyroxine replacement therapy was initiated after surgery, this axis was classified as deficient at follow-up.

### Data acquisition

Measurements of GFAP, NfL, and tau were available for the 64 patients who enrolled in the study after October 2017. Blood samples were collected the day before surgery and on postoperative days 1 and 5. GFAP, NfL, and tau concentrations were analyzed by board-certified laboratory technicians, blinded to the clinical data, using commercially available immunoassays (Quanterix, Billerica, MA, USA) on an HD-1 Analyzer (Quanterix, Billerica, MA, USA). All samples were analysed simultaneously using a single batch of reagents. Baseline and follow-up samples from the same individual were analyzed side by side on the same plates. Intra-assay coefficients of variation were < 10% according to calculations using internal control samples.

IOH was defined as the duration (in minutes) spent below a relative threshold of 20% below a MAP defined as the lowest pressure recorded the day before surgery or before anesthesia delivery, or an absolute threshold of 65 mmHg. Blood pressure measurements were extracted from the anesthesia records at 5-min intervals starting at arrival in the operating room and continuing until the end of general anesthesia.

All patients underwent preoperative gadolinium-enhanced magnetic resonance imaging using T1-weighted sequences on a 3 T Achieva dStream system (Philips, Amsterdam, Netherlands). Experienced neuroradiologists assessed hypothalamic involvement defined as radiological evidence of contact, compression, or displacement of the hypothalamus, as well as compression of the optic chiasm, both of which were subsequently classified as either present or absent.

### Statistical analysis

Associations between biomarker concentrations and new APH-D and AVP-D, respectively, were assessed using mixed-effects models. Separate models were specified for each biomarker and outcome (i.e., six models covering three biomarkers and two outcomes), where the response variable was the log-transformed biomarker concentration. The explanatory variables were log-transformed baseline concentration, time of measurement as a categorical variable (day 1 or 5), and a categorical variable indicating new deficiency (APH-D or AVP-D). Models were evaluated through residual plots, assessment of unmodeled residual correlation, and identification of influential observations through Cook’s distance and standardized changes in coefficients by omitting individual observations (DFBETAS). Missing biomarker measurements were handled by omitting individual measurements rather than removing all measurements for a single individual. Sensitivity analyses were conducted by calculating post-estimation cluster-robust standard errors, which accommodate unmodeled residual correlation and heteroscedasticity within clusters. Additionally, a secondary analysis using Mann–Whitney *U* tests was performed to compare biomarker concentrations on days 1 and 5 between individuals with and without new APH-D and AVP-D, respectively.

Association between IOH and new APH-D or AVP-D was explored using Mann–Whitney *U* tests. Logistic regression was then applied to assess the robustness of any associations and adjust for potential confounding. For the adjusted analyses, we considered potential common causes of IOH, such as surgery duration, reoperation, and blood loss, and new deficiencies. We considered surgical duration as the most critical variable to control for, given that the sample size limited the number of explanatory variables. Surgical duration can be assumed to both increase time at risk for hypotension and the probability of structural injury, as it may be associated with other confounders, such as blood loss. We planned additional adjustments by adding other confounders to the model in the event that IOH remained significant after adjusting for surgical duration. Model diagnostics included assessing influential observations through Cook’s distance, leverage, and studentized residuals, as well as evaluating model misspecification using DHARMa residuals [17].

Because individuals with pre-existing APH-D may present a different risk of developing an additional deficiency [18], we also conducted a Fisher’s exact test to evaluate this association.

Standardized mean differences (SMDs) were used to describe differences in baseline characteristics between participants with and without new deficiencies. SMDs quantify the magnitude of group differences and are considered a more informative measure of imbalance than multiple hypothesis tests, which can be misleading due to their dependence on sample size [19]. For continuous variables, SMDs are calculated as the standardized difference in means. For categorical variables, SMDs are based on the difference in group proportions, standardized by the average of the group-specific binomial variances. Values between 0.1 and 0.5 are typically considered indicative of a small-to-moderate imbalance. A *p* < 0.05 was considered statistically significant. No adjustment for multiple comparisons was performed. All analyses were performed using R statistical software (v4.4.2; R Core Team 2025).

## Results

The study enrolled 100 participants, all of whom underwent ETS. None of the participants received radiotherapy prior to follow-up. Five individuals were excluded due to preoperative panhypopituitarism. Biomarker data were available for 64 participants, providing 183 measurements for GFAP (4% missing) and 187 measurements for tau and NfL (4% missing). Those who developed new deficiencies had fewer biomarker samples available at each time point (*n* = 13–15 for new APH-D; *n* = 5–7 for new AVP-D) as compared with those not developing new deficiencies (*n* = 33–37 and *n* = 52–57, respectively; see the Online Resource).

Study participants had a median age of 65 years (interquartile range [IQR] 54–73) and were predominantly male (*n* = 65; 69%), with 45% of the cohort also presenting preoperative hypertension (*n* = 42). Most patients (*n* = 86; 91%) received sevoflurane and remifentanil for anesthesia maintenance, with continuous norepinephrine infusion used in 94% of procedures. SMDs identified several small-to-moderate imbalances in baseline and intraoperative characteristics between individuals with and without new APH-D and AVP-D (Table 1).

**Table 1:** Baseline patient, adenoma, and intraoperative variables

Characteristic	Overall *n* = 95	New APH-D		SMD^a^	New AVP-D		SMD^a^
		Yes *n* = 24	No *n* = 71		Yes *n* = 12	No *n* = 83	
Gender				0.44			0.07
Male	66 (69%)	13 (54%)	53 (75%)		8 (67%)	58 (70%)	
Female	29 (31%)	11 (46%)	18 (25%)		4 (33%)	25 (30%)	
Age, y	65 (54–73)	66 (54–75)	65 (54–73)	0.07	66 (50–74)	65 (54–73)	−0.12
BMI, kg/m^2^	26.8 (24.3–30.2)	27.7 (24.4–32.8)	26.7 (24.3–29.6)	0.40	28.7 (24.7–36.2)	26.7 (24.3–29.6)	0.58
ASA class				0.37			0.34
I	17 (18%)	5 (21%)	12 (17%)		2 (17%)	15 (18%)	
II	61 (64%)	17 (71%)	44 (62%)		9 (75%)	52 (63%)	
III	17 (18%)	2 (8.3%)	15 (21%)		1 (8.3%)	16 (19%)	
Repeated surgery	23 (24%)	6 (25%)	17 (24%)	0.02	4 (33%)	19 (23%)	0.23
Hypertension	42 (44%)	14 (58%)	28 (39%)	0.38	5 (42%)	37 (45%)	−0.06
Chiasmal compression	84 (88%)	22 (92%)	62 (87%)	0.14	10 (83%)	74 (89%)	−0.17
Hypothalamic involvement^b^	13 (14%)	7 (29%)	6 (8.5%)	0.55	3 (25%)	10 (12%)	0.34
Blood loss, mL	100 (50–200)	200 (30–250)	100 (50–200)	0.30	175 (55–275)	100 (50–200)	0.39
Anesthesia method				0.56			0.46
Sevoflurane/remifentanil	87 (92%)	21 (88%)	66 (93%)		12 (100%)	75 (90%)	
Propofol/remifentanil	4 (4.2%)	0 (0%)	4 (5.6%)		0 (0%)	4 (4.8%)	
Sevoflurane/fentanyl	4 (4.2%)	3 (13%)	1 (1.4%)		0 (0%)	4 (4.8%)	
Surgery duration, h	3.0 (2.5–3.7)	3.0 (2.6–4.0)	3.0 (2.5–3.7)	0.30	3.4 (2.8–4.4)	3.0 (2.5–3.7)	0.51
Norepinephrine, µg (cumulative)	1082 (558–1527)	1138 (447–1541)	1082 (578–1515)	−0.01	860 (331–1271)	1112 (578–1554)	−0.41

Values are presented as *n* (%) or median (IQR).

^a^ SMDs are presented to quantify baseline differences between groups, where SMDs between 0.1 and 0.5 indicate small-to-moderate imbalance.

^b^ Hypothalamic involvement is defined as radiological evidence of contact, compression, or displacement of the hypothalamus.

Abbreviations: *ASA* american society of anesthesiologists physical status classification, *APH-D* anterior pituitary hormone deficiency, *AVP-D* arginine vasopressin deficiency, *BMI* body mass index, *IQR* interquartile range, *SMD* standardized mean difference

Before surgery, 19 individuals (20%) presented both corticotropic and thyrotropic deficiencies, whereas none of the participants presented AVP-D. Thus, analysis of associations between biomarkers, IOH, and APH-D was limited to 76 individuals, with 95 individuals available for analysis of associations with AVP-D.

At 12-months postsurgery, 24/76 individuals (32%) had developed a new APH-D, and 12/95 (13%) had developed a new AVP-D. Among those with a new APH-D, 4/76 (5%) presented a new isolated corticotropic deficiency, 13/76 (17%) a new isolated thyrotropic deficiency, and 7/76 (9%) had developed both deficiencies. Only three individuals developed a new AVP-D without also developing a new APH-D. Those with one APH-D at baseline did not differ in their probability of developing a second APH-D relative to those without any APH-D at baseline (Fisher’s exact test; *p* = 1.0).

### Associations between biomarkers and new deficiencies

**Fig. 1:**
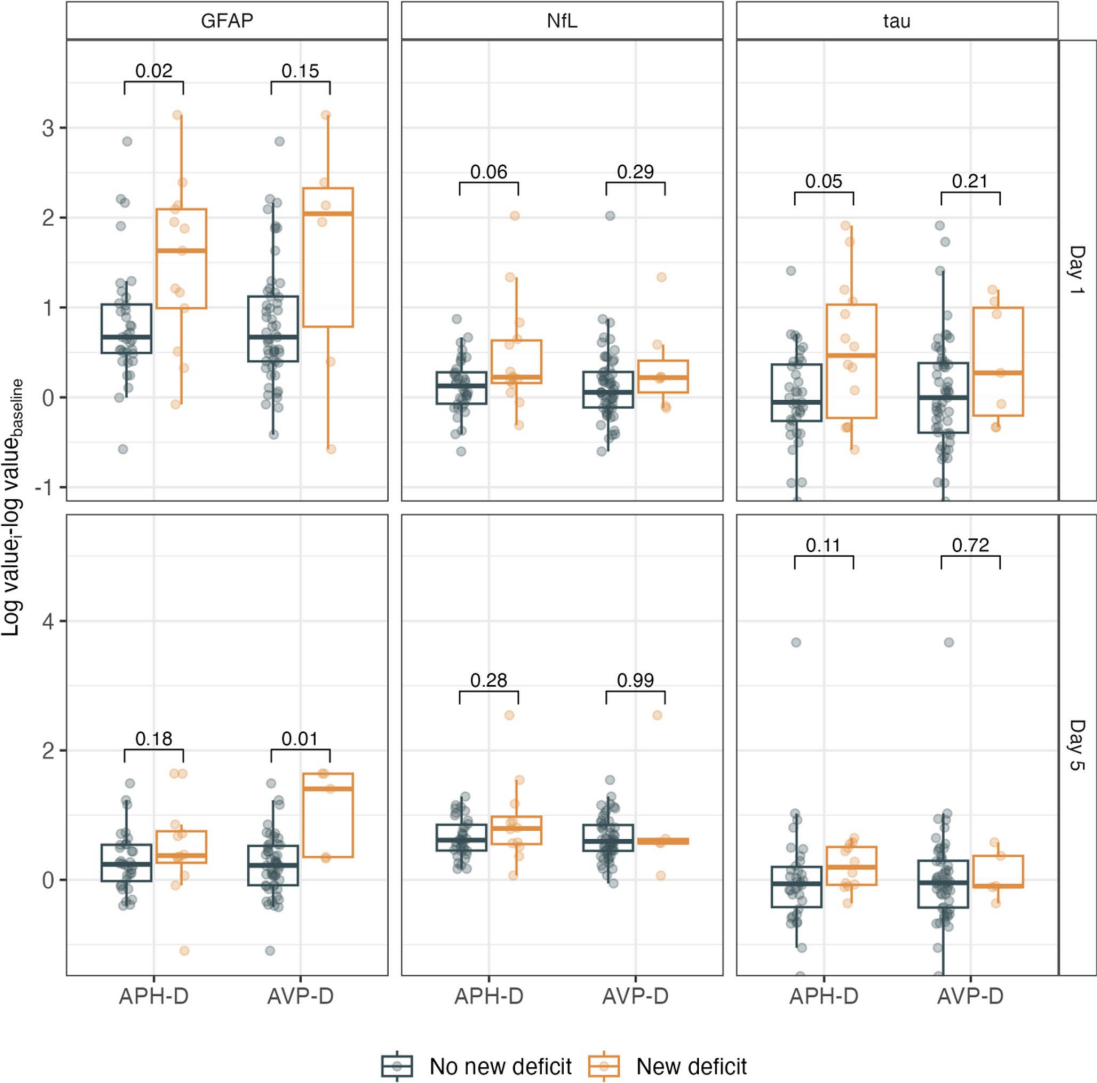
Boxplots show differences in log-transformed GFAP, NfL, and tau on postoperative days 1 and 5 from baseline at 12-months postsurgery according to new APH-D or new AVP-D. P-values are derived from Mann–Whitney *U* tests at each time point. Although increases in biomarker concentrations were higher among individuals experiencing both new APH-D and new AVP-D, these changes were only statistically significant for GFAP and tau on day 1 (new APH-D) and GFAP on day 5 (new AVP-D). Abbreviations: APH-D, anterior pituitary hormone deficiency; AVP-D, arginine vasopressin deficiency; GFAP, glial fibrillary acidic protein; NfL, neurofilament light chain

Absolute and relative biomarker concentrations on days 1 and 5 were higher among individuals who developed a new APH-D. For those with a new AVP-D, all absolute and relative biomarker concentrations were also higher on day 1 but not on day 5 (Table 2). Mann–Whitney *U* tests revealed statistical significance for GFAP on day 5 (new AVP-D) and tau and GFAP on day 1 (new APH-D; Fig. 1).

All biomarkers were associated with the development of new APH-D according to the mixed models, with GFAP showing the largest effect size. By contrast, only GFAP was associated with new AVP-D (Table 3). Influential observations were present in all models, and residual heteroscedasticity was noted in the GFAP model. Consequently, the sensitivity analysis using cluster-robust standard errors showed that the associations between NfL and APH-D and between GFAP and AVP-D, were no longer statistically significant (*p* = 0.12 and *p* = 0.06, respectively). The coefficients in the GFAP and tau models for APH-D remained significant (*p* = 0.04 and *p* = 0.03, respectively).

**Table 2:** Biomarker concentrations and change from baseline at each measurement time point

Time	New APH-D		*P*	New AVP-D		*P*
	Yes (*n* = 13–15)	No (*n* = 34–37)		Yes (*n* = 5–7)	No (*n* = 53–57)	
Absolute concentration (pg/mL)						
GFAP						
Baseline	140.0 (98.9–179.0)	121.0 (95.7–169.8)	0.67	149.5 (124.2–193.5)	121.0 (96.3–182.0)	0.24
Day 1	494.0 (308.0–1285.0)	271.5 (203.0–401.8)	**0.01**	1118.0 (618.0–3358.5)	297.5 (192.8–455.2)	**0.02**
Day 5	198.0 (96.1–302.0)	145.0 (102.0–264.0)	0.50	822.0 (199.0–836.0)	153.0 (96.1–264.0)	**0.02**
NfL						
Baseline	12.5 (8.4–18.9)	15.8 (11.3–20.8)	0.16	12.4 (8.6–17.9)	15.8 (11.6–21.0)	0.21
Day 1	18.9 (14.3–27.2)	18.2 (13.1–24.6)	0.82	20.7 (10.9–22.8)	18.2 (13.1–26.8)	0.73
Day 5	35.7 (19.1–45.3)	31.6 (24.9–42.8)	0.88	42.9 (15.4–45.3)	30.9 (23.9–42.4)	0.95
tau						
Baseline	3.1 (2.6–4.5)	4.1 (2.9–5.3)	0.51	2.8 (2.2–3.4)	4.1 (2.9–5.4)	0.14
Day 1	4.7 (3.7–6.4)	3.3 (2.5–5.2)	0.08	4.2 (2.8–6.4)	3.9 (2.5–5.6)	0.69
Day 5	4.4 (2.7–5.3)	3.1 (2.4–4.4)	0.16	3.0 (2.8–4.4)	3.5 (2.5–4.9)	1.00
Change from baseline (ratio)^a^						
GFAP						
Day 1	5.1 (2.7–8.1)	2.0 (1.6–2.8)	**0.02**	7.7 (2.9–10.3)	2.0 (1.5–3.1)	0.15
Day 5	1.5 (1.3–2.1)	1.3 (1.0–1.7)	0.18	4.1 (1.4–5.2)	1.2 (0.9–1.7)	**0.01**
NfL						
Day 1	1.3 (1.2–1.9)	1.1 (0.9–1.3)	0.06	1.2 (1.1–1.5)	1.1 (0.9–1.3)	0.29
Day 5	2.2 (1.7–2.7)	1.8 (1.6–2.3)	0.28	1.8 (1.8–1.9)	1.8 (1.6–2.3)	0.99
tau						
Day 1	1.6 (0.8–2.8)	0.9 (0.8–1.4)	**0.05**	1.3 (0.8–2.7)	1.0 (0.7–1.5)	0.21
Day 5	1.2 (0.9–1.7)	0.9 (0.7–1.2)	0.11	0.9 (0.9–1.4)	1.0 (0.7–1.3)	0.72

Values are presented as median (IQR). Significant p-values are shown in bold.

^a^ Calculated as the ratio of concentrations on day 1 or 5 to baseline. *P-*values are derived from Mann–Whitney *U* tests.

Abbreviations: *APH-D* anterior pituitary hormone deficiency, *AVP-D* arginine vasopressin deficiency, *GFAP* glial fibrillary acidic protein, *IQR* interquartile range, *NfL* neurofilament light chain

**Table 3:** Fixed-effects estimates from mixed models assessing the association between biomarker concentrations, new APH-D, and new AVP-D

	NfL model		GFAP model		tau model	
Term	Estimate^a^	*P*	Estimate^a^	*P*	Estimate^a^	*P*
APH-D						
Intercept	0.8 (0.34 to 1.31)	0.001	1.3 (−0.28 to 2.89)	0.11	0.4 (−0.00 to 0.86)	0.05
Baseline	0.8 (0.59 to 0.92)	< 0.001	0.9 (0.59 to 1.24)	< 0.001	0.7 (0.40 to 0.99)	< 0.001
Day 5	0.5 (0.38 to 0.65)	< 0.001	−0.7 (−0.90 to −0.44)	< 0.001	−0.1 (−0.28 to 0.17)	0.60
New deficiency	**0.2** **(0.01 to 0.43)**	**0.04**	**0.5** **(0.10 to 0.81)**	**0.01**	**0.4** **(0.02 to 0.71)**	**0.04**
AVP-D						
Intercept	0.9 (0.43 to 1.32)	< 0.001	1.7 (0.49 to 2.85)	0.007	0.5 (0.14 to 0.91)	0.009
Baseline	0.7 (0.58 to 0.89)	< 0.001	0.8 (0.59 to 1.07)	< 0.001	0.7 (0.39 to 0.91)	< 0.001
Day 5	0.5 (0.41 to 0.64)	< 0.001	−0.6 (−0.80 to −0.37)	< 0.001	0.0 (−0.24 to 0.16)	0.69
New deficiency	**0.2** **(−0.11 to 0.44)**	**0.25**	**0.8** **(0.34 to 1.24)**	**< 0.001**	**0.1** **(−0.30 to 0.58)**	**0.52**

Estimates for new deficiencies are presented in bold.

^a^ Estimates represent change in log-transformed biomarker concentration (pg/mL) with 95% confidence intervals.

Abbreviations: *APH-D* anterior pituitary hormone deficiency, *AVP-D* arginine vasopressin deficiency, *GFAP* glial fibrillary acidic protein, *NfL* neurofilament light chain

### Difference in IOH for those with and without new deficiency

**Fig. 2:**
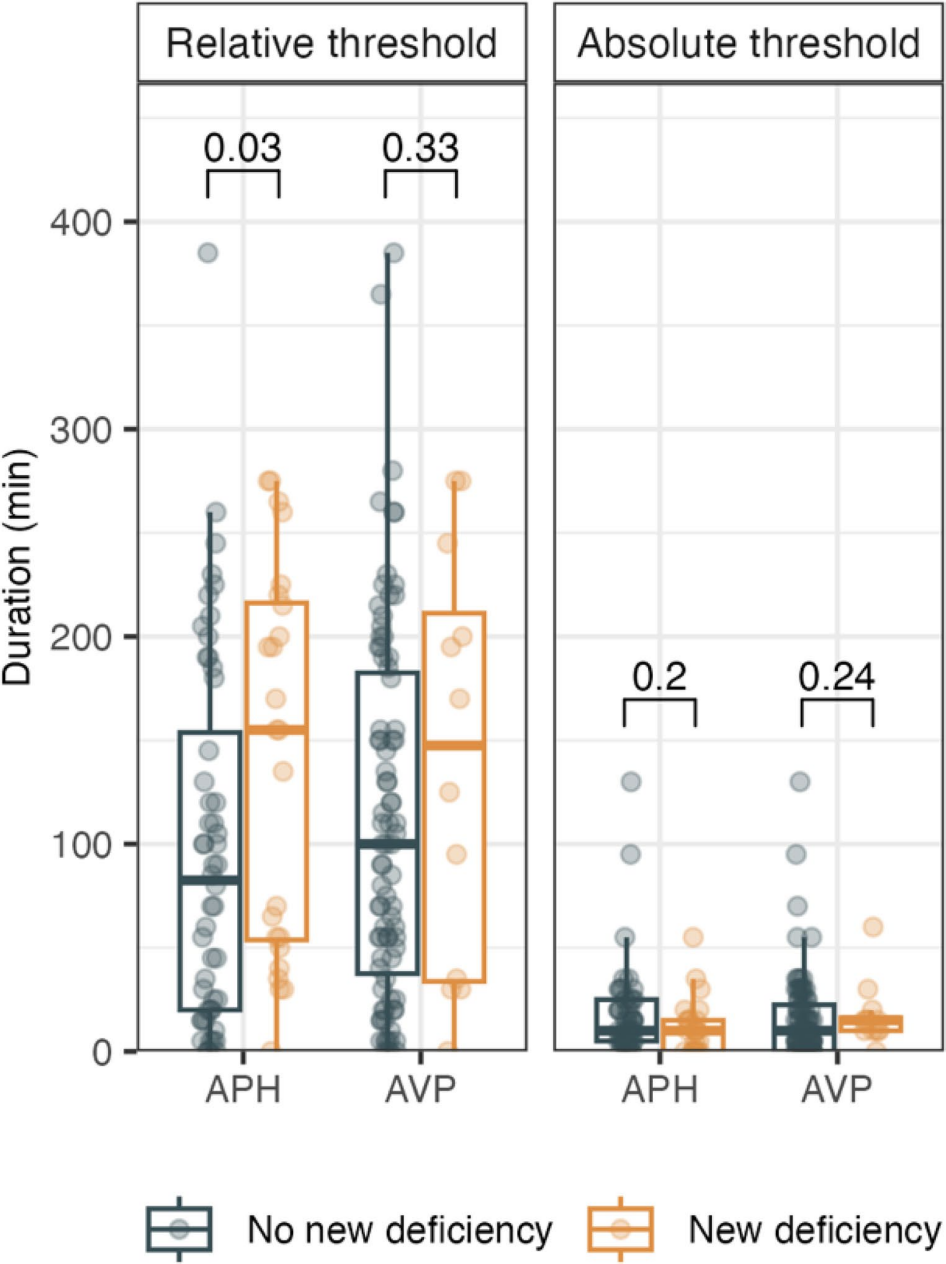
Boxplots show the duration of IOH, defined by a relative threshold (≥20% decrease below preoperative MAP), and an absolute threshold (<65 mmHg). Data are stratified by the presence or absence of new APH-D or new AVP-D at 12-months postsurgery. *P*-values are derived from the Mann–Whitney *U* test. Patients who developed new APH-D experienced longer relative, but not absolute, IOH. No significant difference was observed for those who developed new AVP-D. Abbreviations: APH-D, anterior pituitary hormone deficiency; AVP-D, arginine vasopressin deficiency; MAP, mean arterial pressure

The median (IQR) MAP during surgery was 75.3 mmHg (72.2–78.2), with no participant presenting either median or mean MAP values <65 mmHg. The duration of relative IOH was significantly longer in individuals who developed new APH-D as compared with those who did not (155 min [54–216] vs. 82 min [20–154]; p = 0.03). No significant difference in relative IOH was observed for new AVP-D (148 min [34–211] vs. 100 min [38–182]; p = 0.33; Fig. 2).

We observed no differences in the duration of absolute IOH for either outcome (APH-D: median [IQR], 10 min [0–15] vs. 10 min [5–25]; *p* = 0.22; AVP-D: 15 min [10–16] vs. 10 min [0–22]; *p* = 0.24). Overall, the duration of relative IOH was substantially longer than that of absolute IOH (100 min [35–190] vs. 10 min [5–20]).

In adjusted logistic regression analysis (including surgery duration), relative IOH was not significantly associated with new APH-D (aOR 1.005, 95% CI 0.998–1.012; *p* = 0.15) or new AVP-D (aOR 1.000, 95% CI 0.992–1.008; *p* = 0.98). Unadjusted analysis likewise showed no statistically significant associations between relative IOH and new APH-D (OR 1.005, 95% CI 1.000–1.011; *p* = 0.054) or new AVP-D (OR 1.003, 95% CI 0.997–1.010; *p* = 0.30).The APH-D model was sensitive to a single outlier with markedly elevated IOH but no new deficiency, and outlier removal resulted in a statistically significant association. As the adjusted models showed no significant association between IOH and outcomes, further adjustment for additional confounding was not pursued.

## Discussion

This study evaluated factors associated with new pituitary hormone deficiencies after ETS for NFPAs. We found that elevated postoperative GFAP, NfL, and tau were associated with new APH-D, with changes in GFAP also associated with new AVP-D. Additionally, we found that relative IOH was weakly associated with new APH-D but not new AVP-D.

The different associations of brain injury biomarkers with each respective new deficiency suggest that the underlying mechanisms of injury may differ and potentially involve distinct hormone-regulating structures. Previous studies report GFAP concentrations in the posterior pituitary and, to a lesser extent, the anterior pituitary [20], supporting the possibility that direct pituitary injury may contribute to its release. By contrast, the presence of NfL and tau in the pituitary gland is less established [21], and their release may not result from direct pituitary damage in the same way as GFAP. Associations between APH-D and elevated brain injury biomarker concentrations may instead reflect injury to hypothalamic structures that regulate anterior pituitary function. However, this interpretation remains speculative, and the findings could also be explained by collateral injury to nearby neural structures not directly involved in hormonal control.

Given its association with both new APH-D and new AVP-D, GFAP may be a more useful predictor of new pituitary hormone deficiencies following ETS for NFPA than tau or NfL. However, this interpretation should be regarded with caution given the limited sample size. We also only measured NfL up to postoperative day 5. Because NfL can continue to increase for at least 10 days following neurological surgery [22], it is possible that measurements taken at later time points might have revealed a stronger association.

The present data do not permit reliable identification of the optimal sampling time for prediction of new pituitary deficiencies. Nevertheless, relative increases in GFAP and tau were greater on day 1 among patients who developed new deficiencies, suggesting that earlier postoperative sampling may be more informative for these biomarkers. This interpretation is consistent with prior findings demonstrating higher plasma tau and GFAP levels on day 1 compared with day 5 [9].

Relative IOH was higher in those who developed new APH-D but not in those with new AVP-D. Choi et al. reported a higher risk of developing postoperative adrenal insufficiency in patients undergoing NFPA surgery who were anesthetized with sevoflurane compared to propofol [23]. In their study, the sevoflurane group also experienced more IOH, suggesting potential confounding by anesthetic technique and supporting a possible link between IOH and new APH-D. It may therefore be hypothesized that maintaining intraoperative blood pressure closer to resting levels could reduce the risk of new APH-D. Since the vast majority of patients in our cohort (95%) were anesthetized with sevoflurane, the observed association with IOH is unlikely to be explained by choice of anesthetic agent.

Our findings further suggest that the anterior pituitary may be more vulnerable to IOH than the posterior pituitary. However, although absolute IOH was not significantly associated with AVP-D, the duration of relative IOH was longer among patients with new AVP-D. The absence of a statistically significant difference may therefore reflect limited statistical power.

No differences were observed in absolute IOH for either outcome in our cohort. This may reflect the high mean MAP maintained during all procedures, with periods of MAP < 65 mmHg being brief and likely unintentional. One possible interpretation of this finding is that sustained periods of moderately reduced blood pressure (above 65 mmHg but below a relative threshold) may be more detrimental to anterior hormone-regulating structures than short episodes of more severe hypotension.

We found no statistically significant association between relative IOH and new APH-D in the adjusted analysis. The effect of IOH may therefore be confounded by longer and more complex procedures, which increase the probability of accumulating time below a given MAP threshold. However, adjusting for surgical duration may be overly conservative, as time spent below a critical perfusion threshold is a key determinant of hypoperfusion-related injury. Disentangling the independent contribution of hypotension, while also accounting for other potential confounding, would require a substantially larger sample than was available in this cohort.

This study has several strengths. It is the first to report an association between postoperative hormone deficiencies and specific brain injury biomarkers, as well as a potential link to IOH. The study was also conducted at a high-volume centre treating a broad spectrum of pituitary disorders. Both surgical procedures and endocrine evaluations were standardized and performed by three experienced pituitary neurosurgeons and a team of experienced endocrinologists. All endocrinologic samples were collected, processed, and analysed in a single laboratory, ensuring consistency. We also employed mixed models to leverage the available data and performed several sensitivity analyses to assess and report the robustness of our findings. Finally, the observed frequencies of new hormone deficiencies align with previously reported rates, which enhances generalizability [6, 24, 25].

This study has several limitations, the most significant of which involves the small sample size. Although the mixed-effects models demonstrated associations between the biomarkers and new deficiencies, these were not consistently observed in the Mann–Whitney *U* tests. This may reflect the greater statistical efficiency of the mixed models, which leverage repeated measures and thus incorporate more information. However, estimating robust standard errors in both the GFAP model for new AVP-D and the NfL model for new APH-D altered the statistical significance of the coefficients. Additionally, influential observations were identified in all models. This is at least partially attributable to the limited sample size, as biomarker estimates were based on a small number of individuals. However, variability in the statistical significance of the estimates using robust standard errors and the presence of influential observations emphasize that these results should be hypothesis-generating and require confirmation in larger cohorts.

Similarly, the association between relative IOH and new APH-D lacked statistical significance in both adjusted and unadjusted logistic regression models, further emphasizing the uncertainty of these results and the need for replication in larger cohorts. Furthermore, we restricted our analyses to NFPAs, which reduces the generalizability of the findings to other pituitary neoplasms, such as functioning adenomas and craniopharyngiomas. The data also do not permit assessment of the clinical impact of new APH-D, given that thyrotropic deficiency is easier to manage and incurs less morbidity than corticotropic deficiency.

The mixed-effects models used to investigate the association between biomarkers and outcomes were not intended to estimate the probability of new pituitary deficiencies, in contrast to classification approaches such as logistic regression. The mixed-effects approach allowed us to efficiently assess associations between perioperative biomarker trajectories and outcomes without any a priori assumptions regarding the most informative biomarker measure (e.g., absolute value, relative change, or area under the curve). Furthermore, selecting such measures post hoc would have relied on observed data and increased the risk of model overfitting and type I errors. Moreover, the limited number of events in this cohort further supports the choice of the mixed-effects models, as any classification model would likely lead to imprecise and unstable estimates.

The present study was designed to address associations with long-term pituitary deficiencies. We did not evaluate early postoperative AVP-D, which is often transient but still clinically relevant. Exploring whether early biomarker elevations predict transient AVP-D could provide additional value, as biomarkers might serve as early warning signals to identify high-risk patients and prioritize surveillance. Evaluating such associations represents a possible direction for future research.

Finally, estimating the amount of relative hypotension requires valid measurements of reference blood pressures. Because pre-induction blood pressure alone is insufficient to establish the normal range [26], we also measured resting blood pressure the day before surgery. However, it is possible that we did not adequately capture the resting blood pressure of each individual in the cohort.

## Conclusion

Postoperative elevations in circulating GFAP, NfL, and tau were associated with new APH-D, while elevated GFAP was also linked to new AVP-D. These biomarkers may serve as early indicators of intraoperative injury associated with clinically relevant endocrine outcomes. Future studies with larger cohorts will be needed to evaluate predictive performance and identify potential cutoff values for perioperative biomarker measurements associated with new AVP-D and APH-D. Although relative IOH was associated with new APH-D, larger observational studies and randomized trials are needed to confirm these findings and determine whether targeting relative intraoperative blood pressure thresholds can reduce the risk of new postoperative pituitary deficiencies.

## Supplementary Information

Below is the link to the electronic supplementary material.

Supplementary Material 1 (DOCX 18.9 KB)

## Data Availability

Data are available from the corresponding author on reasonable request, subject to applicable regulations.
