# Supplementary material for: Brain injury biomarkers and intraoperative hypotension: associations with pituitary hormone deficiency following transsphenoidal endoscopic surgery for non-functioning pituitary adenomas
Source: Pituitary. 2025 Dec 8;29(1):9. doi: 10.1007/s11102-025-01597-y (PMC12686086; doi:10.1007/s11102-025-01597-y)
Supplement: Supplementary file 1 — Supplementary Material 1 (DOCX 18.9 KB) [file 11102_2025_1597_MOESM1_ESM.docx]

**Online Resource**

***Pituitary***

**Brain injury biomarkers and intraoperative hypotension: associations with pituitary hormone deficiency following transsphenoidal endoscopic surgery for non-functioning pituitary adenomas**

Thorsson M, Trimpou P, Asztély M, Hallén T, Hantelius V, Blennow K, Zetterberg H, Johannsson G, Oras J, Skoglund T

**Correspondence**

Martin Thorsson, MD

Department of Anesthesia and Intensive Care

Sahlgrenska University Hospital, Gothenburg, SWEDEN

Email: martin.thorsson@vgregion.se

**Table 1** Number of biomarker measurements available at each time point, stratified by outcome (APH-D, AVP-D) and status (yes/no)

|  | | APH-D | | AVP-D | |
| --- | --- | --- | --- | --- | --- |
| Biomarker | Time | No | Yes | No | Yes |
| GFAP | Baseline | 36 | 13 | 54 | 6 |
| GFAP | Day 1 | 36 | 15 | 56 | 7 |
| GFAP | Day 5 | 32 | 13 | 52 | 5 |
| NfL | Baseline | 37 | 14 | 55 | 7 |
| NfL | Day 1 | 37 | 15 | 57 | 7 |
| NfL | Day 5 | 33 | 13 | 53 | 5 |
| tau | Baseline | 37 | 14 | 55 | 7 |
| tau | Day 1 | 37 | 15 | 57 | 7 |
| tau | Day 5 | 33 | 13 | 53 | 5 |

Abbreviations: APH-D, anterior pituitary hormone deficiency; AVP-D, arginine vasopressin deficiency; GFAP, glial fibrillary acidic protein; NfL, neurofilament light chain.
